# A Deceiving Massive Opacification of the Maxillary Sinus: Odontogenic Cyst Mimicking Odontogenic Sinusitis

**DOI:** 10.1002/ccr3.72840

**Published:** 2026-06-01

**Authors:** Anastasia Urbanelli, Giuseppe Riva, Giancarlo Pecorari, Giovanni Felisati, Alberto Maria Saibene

**Affiliations:** ^1^ Otorhinolaryngology Unit, Department of Surgical Sciences University of Turin Turin Italy; ^2^ Otorhinolaryngology Unit, Department of Health Sciences, Santi Paolo e Carlo Hospital University of Milan Milan Italy

**Keywords:** dentigerous cyst, maxillary sinus, nasal endoscopy, odontogenic cyst, odontogenic sinusitis, sinus opacification

## Abstract

Considered the second most common type of odontogenic cysts, dentigerous cysts (DCs) are developmental lesions most often detected incidentally on radiographic imaging. The pathogenesis of DCs is not fully understood, but they are widely regarded as developmental cysts originating from the dental follicle. Although typically asymptomatic, DCs may cause clinical symptoms when they reach large sizes or get infected. This case highlights how a DC (common developmental odontogenic lesion often incidentally detected on imaging) may present with complete maxillary sinus opacification despite minimal symptoms and normal endoscopic findings, underscoring the need for precise terminology when classifying odontogenic‐related sinonasal conditions.

## Key Clinical Message

Dentigerous cysts involving the maxillary sinus may radiologically mimic odontogenic sinusitis despite minimal symptoms and normal nasal endoscopy. Distinguishing true inflammatory ODS from noninflammatory odontogenic lesions is essential to avoid misclassification, inappropriate sinonasal interventions, and ensure accurate diagnosis and patient safety.

## 1 Introduction

Cystic lesions of the orofacial region encompass a broad spectrum of pathologic entities of both odontogenic and non‐odontogenic origin [1]. Among these, odontogenic cysts, which are defined by the accumulation of fluid within a cavity, are among the most frequently encountered lesions involving the oral and maxillofacial structures [2]. Their pathogenesis is largely attributed to the activation and proliferation of residual odontogenic epithelial remnants entrapped within the jawbones. Dentigerous cysts (DCs) are odontogenic cysts arising in association with the crowns of unerupted teeth. They result from the expansion of the dental follicle and are typically attached to the cementoenamel junction [3]. DCs occur predominantly in the mandible, representing more than 70% of reported cases, and show a marked male predominance, with a male‐to‐female ratio exceeding 2:1. They are rarely observed in the pediatric population [4].

Clinically, DCs are usually asymptomatic and are often detected incidentally as well‐circumscribed radiolucent lesions surrounding the crown of an unerupted tooth—most commonly the mandibular third molar—on panoramic radiographs obtained during routine dental examinations or in the evaluation of delayed tooth eruption. Histopathologically, DCs are lined by nonkeratinized stratified squamous epithelium, analogous to reduced enamel epithelium [5]. Treatment strategies for DCs are influenced by factors such as cyst size, anatomical location, and patient age. The conventional management approach consists of surgical enucleation of the cyst, typically combined with extraction of the associated tooth [6].

In this context, large odontogenic cystic lesions involving the maxillary sinus, such as dentigerous cysts, may represent a relevant source of diagnostic ambiguity [7]. Radiologically, these lesions can produce partial or complete maxillary sinus opacification and close spatial relationships with dental structures, features that may erroneously suggest a diagnosis of odontogenic sinusitis when interpreted in isolation [8]. However, the absence of sinonasal inflammatory signs on nasal endoscopy and the frequent lack of overt sinonasal symptoms highlight a fundamental pathophysiological distinction between true odontogenic sinusitis and noninflammatory odontogenic lesions secondarily involving the sinus cavity [9]. Although surgical management may ultimately address both conditions effectively, conflating these entities risks treating a radiological finding rather than a defined disease process. This semantic and diagnostic overlap may lead to unnecessary sinonasal interventions, inappropriate indications, or an underestimation of patient‐specific risks, ultimately impacting clinical decision‐making and patient safety [9]. A precise and consistent use of terminology, supported by the integration of clinical, endoscopic, and radiological findings, therefore remains essential to avoid misclassification and to ensure tailored, evidence‐based management.

We report the case of a young male patient with a large dentigerous cyst involving the maxillary sinus, radiologically presenting as complete sinus opacification in the setting of vague, nonspecific symptoms and a normal nasal endoscopic examination. While dentigerous cysts occupying the maxillary sinus have previously been described, the novelty of the present report lies in its diagnostic implications. Specifically, this case highlights how complete unilateral maxillary sinus opacification alone may be insufficient to support a diagnosis of odontogenic sinusitis in the absence of corresponding clinical and endoscopic evidence of active sinonasal inflammation. This case is presented as a paradigmatic example of how noninflammatory odontogenic lesions may mimic odontogenic sinusitis on imaging alone, thereby contributing to diagnostic and terminological ambiguity. By correlating radiological findings with clinical presentation and endoscopic assessment, this report aims to illustrate the importance of distinguishing true odontogenic sinusitis from odontogenic conditions secondarily affecting the maxillary sinus, reinforcing the need for precise classification to guide appropriate and proportionate management.

## 2 Case History

A 16‐year‐old male student presented to our clinic with a mild pressure sensation in the right maxillary region, below the right eye, persisting for approximately 2 months. He described the symptom not as pain but as a “mild discomfort,” without radiation to the head, jaw, or other facial areas, and reported no impact on daily activities or sleep quality. He denied associated symptoms, including facial pain, dental pain, nasal discharge, postnasal drip, nasal obstruction, or hyposmia.

The patient reported no relevant history of dental complications and had not undergone dental extractions or other dental treatments, such as prosthetic procedures or dental implant placement. He reported regular yearly professional dental cleaning. Apart from a known pollen and grass allergy, he was in good general health, up to date with vaccinations, and not taking any medications. He had never used nasal therapies, as he had no history of nasal symptoms.

**FIGURE 1:**
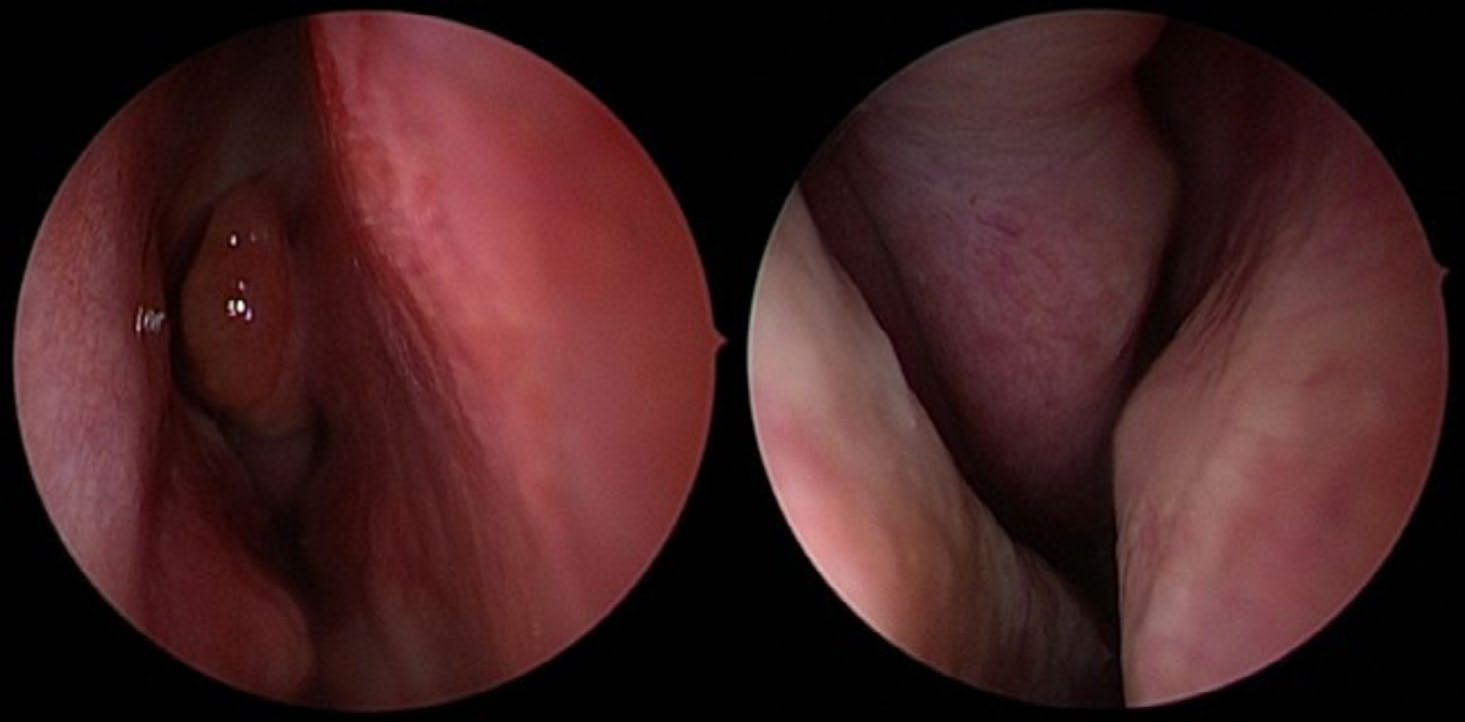
Endoscopic examination of the right nasal cavity. Left: Inferior and middle turbinate can be observed on the right side of the image, and the septum on the left side. No edema, purulence, or polyps are visible. Right: In this close‐up of the right ostiomeatal complex, no edema, purulence, or polyps are visible.

On physical examination, the patient appeared calm and comfortable despite the reported maxillary pressure sensation and was afebrile. Nasal endoscopic examination revealed healthy, normal‐appearing nasal mucosa (see Figure 1). No purulent secretions or nasal polyps were observed, and both nasal cavities were fully accessible for inspection. Oral cavity examination showed preserved dentition with no evidence of dental caries. Palpation of the palatal area corresponding to the right maxillary sinus did not elicit increased discomfort, and sinus trigger points were non‐tender. The oropharynx was unremarkable, and ophthalmological examination revealed no abnormalities.

## 3 Differential Diagnosis, Investigations, and Treatment

**FIGURE 2:**
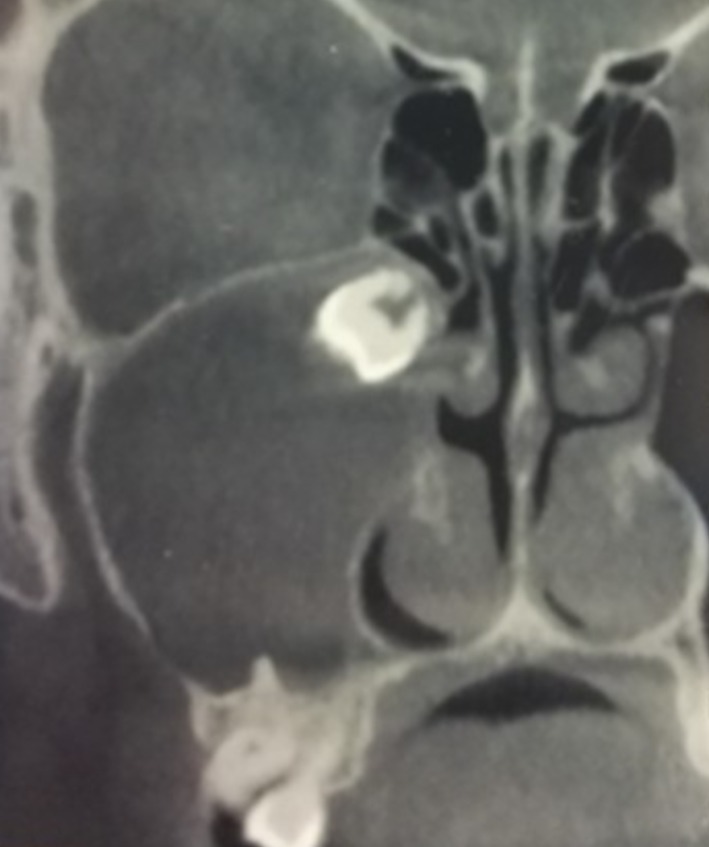
Head computed tomography coronal image showing the dentigerous cysts of the right maxillary sinus, including the tooth germ corresponding to tooth 1.8. A complete opacification of the right maxillary sinus can be observed.

To exclude a possible odontogenic origin of the mild symptoms, and in agreement with the patient's dental provider, radiological evaluation was performed. Computed tomography (CT) revealed a well‐circumscribed, round, fluid‐density lesion within the right maxillary sinus, containing a tooth germ corresponding to tooth 18 (upper right third molar) at its superior aspect, consistent with a follicular (dentigerous) cyst. The lesion caused thinning and displacement of the sinus walls, which appeared largely indistinguishable from the cyst capsule. A defect was observed in the inferolateral wall of the maxillary sinus. The tooth germ was located immediately distal to the sinus ostium, with the superior portion of the cyst protruding into the sinus cavity (see Figure 2). Despite the absence of endoscopic secretions and the minimal symptoms reported, the CT scan demonstrated complete opacification of the right maxillary sinus.

In the present case, no formal multidisciplinary meeting was held. However, a dental consultation was obtained, and radiological findings were interpreted by the treating team in conjunction with standard radiological reports.

**FIGURE 3:**
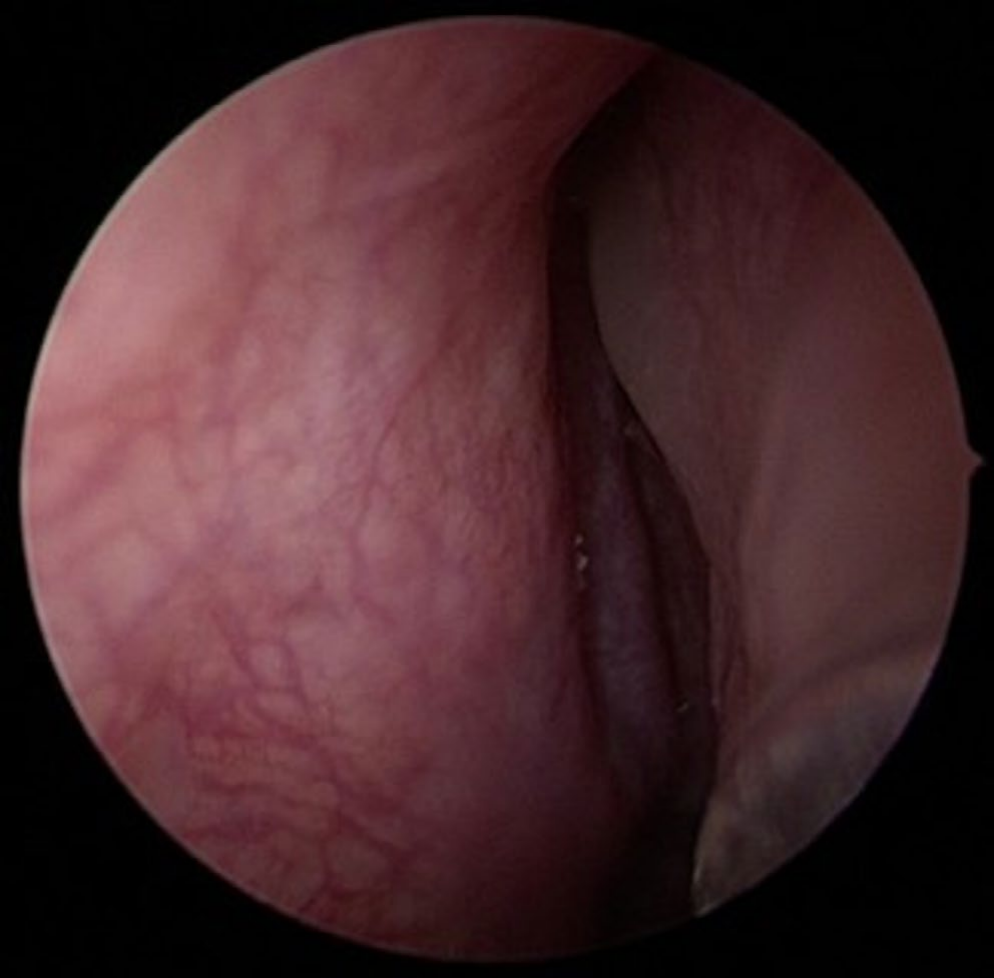
Intraoperative imaging of right osteomeatal complex (free from purulent discharge, edema, or polyps).

**FIGURE 4:**
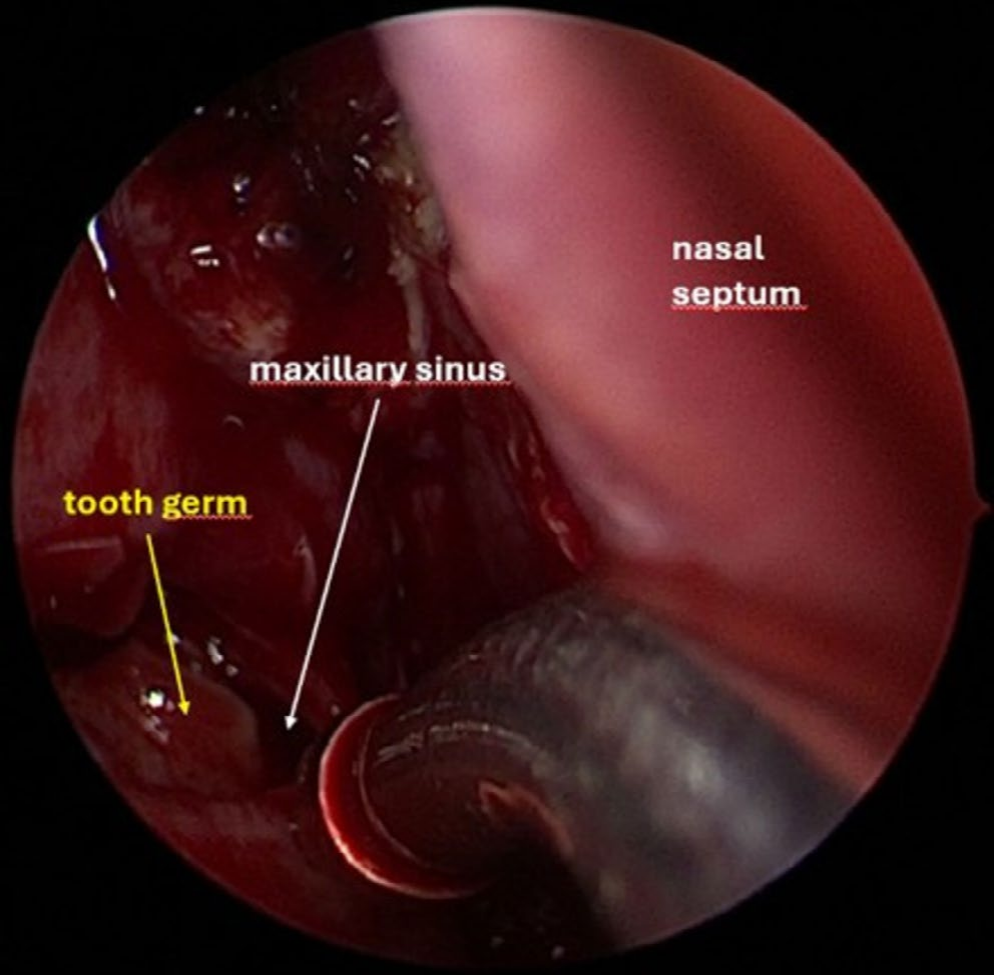
Intraoperative visualization of the tooth germ in the right maxillary sinus, before its removal.

**FIGURE 5:**
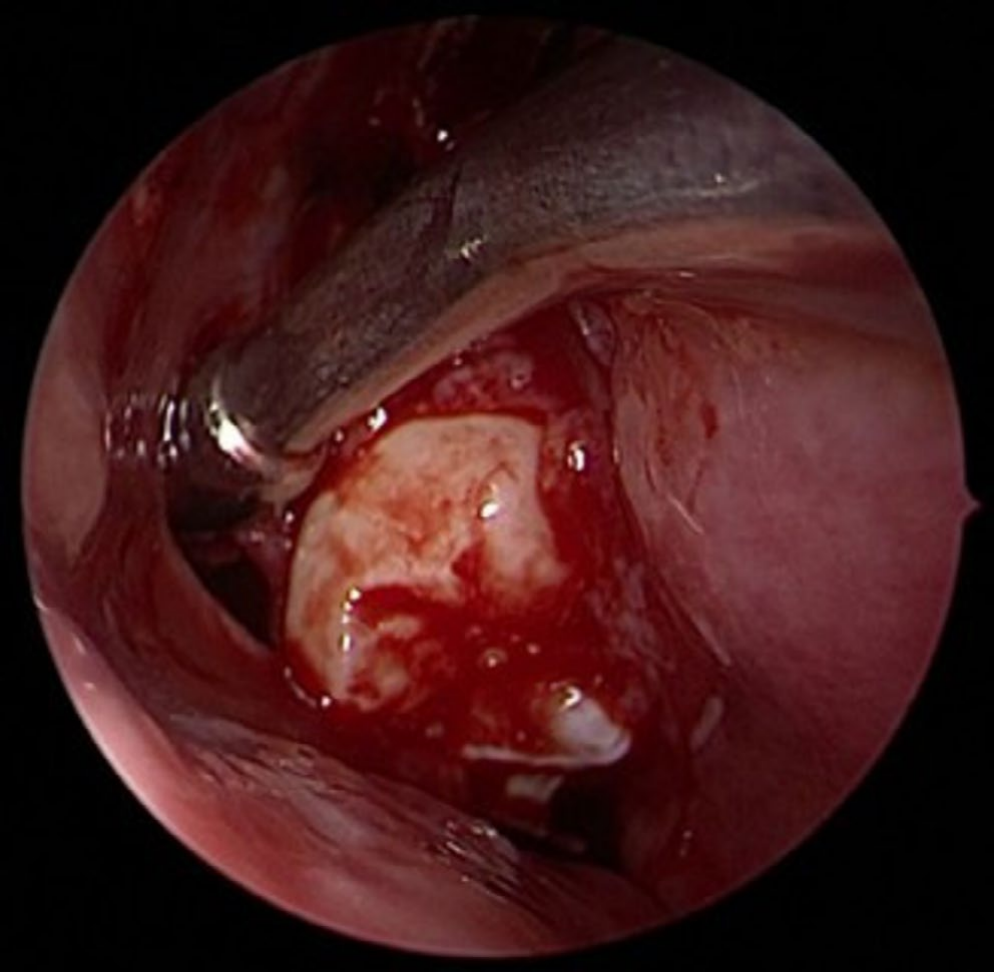
Endoscopic view of the tooth germ in the nasal cavity before complete removal.

**FIGURE 6:**
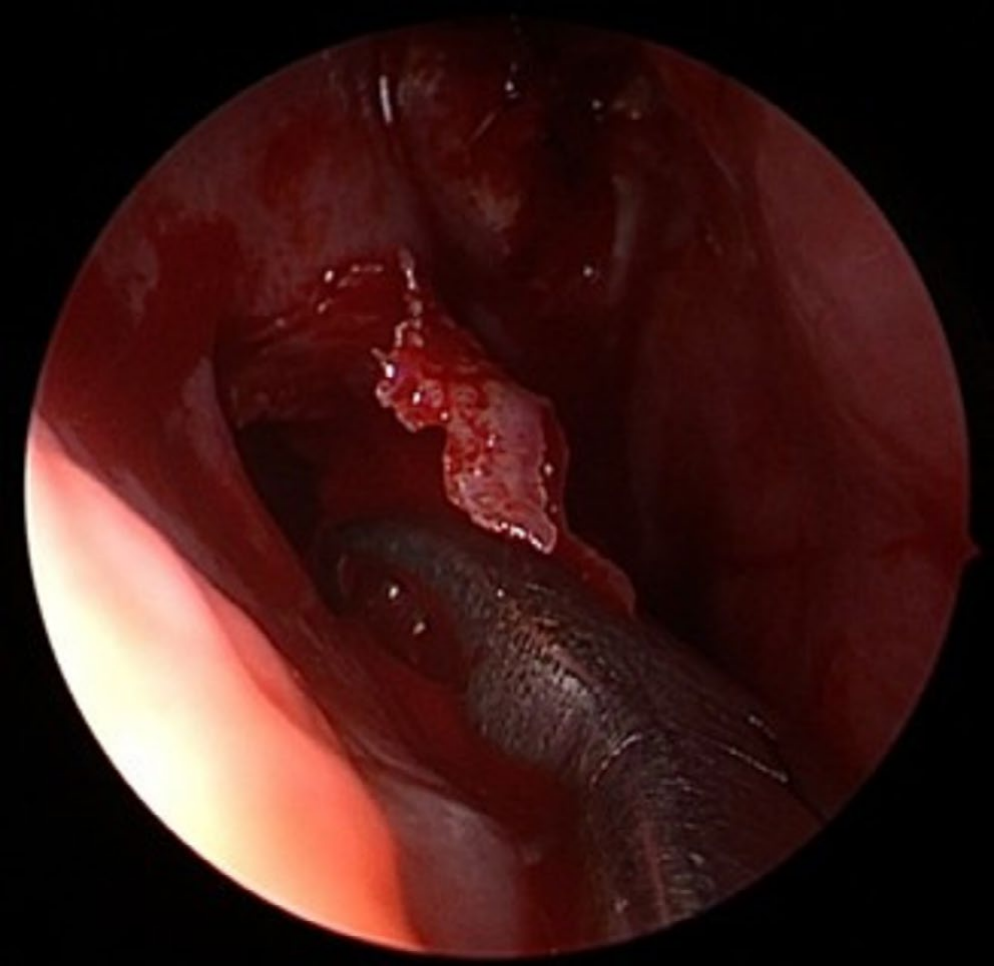
Endoscopic view of the removal of the remainder of the cyst from the maxillary sinus.

Following diagnosis, the patient underwent surgical treatment. Given the position of the tooth germ, an exclusively endoscopic approach was considered the most appropriate surgical strategy. At the beginning of the procedure, no purulent discharge was observed, either at the level of the right osteomeatal complex (see Figure 3) or elsewhere in the nasal cavities or nasopharynx. The middle turbinate was completely removed to facilitate access and removal of the follicular cyst and the associated tooth germ. An uncinectomy and middle meatal antrostomy were then performed. After enlargement of the maxillary sinus ostium, the tooth germ became immediately visible at the sinus opening (see Figure 4). Following extraction of the tooth germ (see Figure 5), the follicular cyst was completely excised (see Figure 6) and sent for histopathological analysis.

## 4 Conclusion and Results

Histopathological examination demonstrated a cystic lesion lined by nonkeratinized stratified squamous epithelium, consistent with a dentigerous cyst. The cyst wall was composed of fibrous connective tissue and showed mild chronic inflammatory infiltrate. Adjacent sinonasal mucosa was also present, with mild chronic inflammation. Postoperatively, the patient was treated with amoxicillin/clavulanic acid for 5 days, in addition to saline nasal irrigations. At follow‐up evaluations conducted up to 6 months postoperatively, the patient consistently reported complete resolution of symptoms and overall well‐being. As early as 15 days after surgery, nasal endoscopy showed no crusting or clots, and the right maxillary sinus appeared well aerated.

## 5 Discussion

Odontogenic sinusitis (ODS) is defined as a maxillary sinusitis of bacterial etiology that arises secondary to dental disease or dental procedures [9]. Traditionally, the diagnosis of ODS ideally involves a dental evaluation confirming the dental focus and an otolaryngological evaluation which confirms the sinus disease with nasal endoscopy [8, 9]. The gold standard for the identification of the dental focus in ODS is CT or cone beam CT (CBCT). For the specific evaluation of dental disease, periapical or panoramic radiographs can also be employed. Clinical diagnosis of ODS is confirmed by nasal endoscopy, which is normally used to assess the middle meatus in order to evaluate the presence of purulent discharge, mucosal edema or polyps [8]. From a comprehensive ODS diagnosis standpoint, the role of CT is limited to the identification of unilateral sinus disease [9], thus inducing suspicion of ODS among other known peculiar clinical features (such as foul smell, foul taste, unilateral purulent discharge, and oral bacteria growth in nasal cultures) [10]. Only in very selected symptomatic and coherent cases, a complete CT opacification *without* purulence in the middle meatus can still be considered supportive of a definitive ODS diagnosis [9].

Therefore, in ODS, CT imaging complements nasal endoscopy by providing, in cases of manifest sinusitis, detailed information on the degree of sinus opacification, the presence of bony erosions, and the involvement of paranasal sinuses beyond the maxillary sinus. In chronic disease, CT may also reveal hyperostosis of the sinus walls or, in the presence of fungal superinfection, the characteristic “iron‐like” signal within the sinus.

However, the term *odontogenic sinusitis* is often used inaccurately to describe conditions in which CT imaging shows maxillary sinus opacification—even when complete—despite a normal nasal endoscopy and minimal or absent clinical symptoms [11]. In the case we report, for instance, the patient presented with complete opacification of the right maxillary sinus, accompanied only by a mild pressure sensation in the right maxillary region and a normal nasal endoscopic examination. In such situations, we assume that the term *odontogenic cyst* is more appropriate than *odontogenic sinusitis*. On the contrary, well‐known instances of symptomatic patients with positive endoscopy and a clear odontogenic focus should always be recognized as ODS, despite mild sinus disease signs on CT scan [12, 13].

This distinction is not merely semantic nor an exercise in excessive diagnostic refinement. The precise use of terminology in odontogenic‐related sinonasal disease is a prerequisite for sound clinical reasoning, appropriate patient management, and meaningful comparison of treatment outcomes across studies. Labeling fundamentally different entities under the same diagnostic umbrella risks conflating inflammatory sinus disease with noninflammatory odontogenic conditions, thereby obscuring pathophysiological mechanisms, diluting therapeutic indications, and compromising the interpretability of the literature. The long‐standing inconsistency in the use of the term “odontogenic sinusitis” has already contributed to heterogeneous study populations, conflicting results, and limited reproducibility. Without rigorous diagnostic definitions grounded in clinical, endoscopic, and radiological correlation, advances in ODS management risk being built on misclassification rather than disease‐specific evidence.

In this context, a multidisciplinary approach involving the otolaryngologist, radiologist, and maxillofacial surgeon may be valuable to ensure accurate interpretation of imaging findings and to optimize diagnostic and therapeutic decision‐making. In particular, oral and maxillofacial radiologists play a key role in the interpretation of CT and CBCT imaging, especially in differentiating odontogenic cystic lesions from other maxillary sinus pathologies, while oral and maxillofacial surgeons are essential in the surgical management and definitive treatment of odontogenic lesions when intervention is required.

Radiological differential diagnosis in this context included odontogenic keratocyst, unicystic ameloblastoma, and sinonasal inflammatory lesions such as mucosal polyps. However, the presence of a well‐defined unilocular radiolucent lesion associated with an impacted tooth germ was most consistent with a dentigerous cyst. Definitive diagnosis was subsequently confirmed by histopathological examination, demonstrating a cystic lesion lined by nonkeratinized stratified squamous epithelium with a fibrous connective tissue wall and mild chronic inflammatory infiltrate, thereby excluding alternative odontogenic and sinonasal pathologies.

From a treatment standpoint, cases not fulfilling the diagnostic criteria for ODS may allow several management options beyond standard endoscopic sinus surgery (ESS). In the absence of endoscopic signs of active sinus disease, treatment may be limited to the management of the odontogenic focus alone, combined with clinical and radiological follow‐up [9, 14, 15]. Conservative strategies, including isolated dental treatment, “watchful waiting,” or short‐course medical therapy (targeted antibiotic therapy against anaerobic odontogenic pathogens, such as amoxicillin–clavulanate, combined with nasal saline irrigations and topical intranasal corticosteroids), have been shown to be effective in selected cases where sinus opacification represents a secondary or noninflammatory odontogenic condition rather than true sinusitis [9, 16, 17] In particular, in the absence of endoscopic signs of active sinusitis, a purely odontogenic approach (consisting in conservative periapical therapy and/or extraction of the causative tooth when indicated) may be sufficient to achieve disease resolution without the need for ESS [9, 14]. However, in the present case, a purely odontogenic approach was not applicable due to the anatomical position of the tooth germ, which was located immediately distal to the maxillary sinus ostium and therefore not accessible through an exclusively trans‐oral dental approach.

This case highlights the importance of integrating clinical evaluation with imaging in the diagnosis and management of odontogenic pathology. Nasal endoscopy, together with a thorough dental and clinical assessment, is essential not only for distinguishing true odontogenic sinusitis from other odontogenic conditions—such as odontogenic cysts—where sinusitis is absent or very mild, ensuring correct use of terminology but also for identifying subtle clinical features that may be entirely missed on imaging alone.

## Author Contributions

**Anastasia Urbanelli:** formal analysis, writing – original draft. **Giuseppe Riva:** data curation, formal analysis, investigation. **Giancarlo Pecorari:** supervision, validation. **Giovanni Felisati:** supervision, validation. **Alberto Maria Saibene:** conceptualization, project administration, writing – review and editing.

## Funding

The authors have nothing to report.

## Disclosure

Permission to reproduce material from other sources: No material was reproduced from other sources.

## Consent

Written informed consent for publishing this report was obtained from the patient in accordance with the journal's patient consent policy.

## Conflicts of Interest

The authors declare no conflicts of interest.

## Data Availability

All data pertaining to this case report are available from the corresponding author upon reasonable request.
